# The Pyrimidine Analog FNC Potently Inhibits the Replication of Multiple Enteroviruses

**DOI:** 10.1128/JVI.00204-20

**Published:** 2020-04-16

**Authors:** Na Xu, Jing Yang, Baisong Zheng, Yan Zhang, Yiming Cao, Chen Huan, Shengqi Wang, Junbiao Chang, Wenyan Zhang

**Affiliations:** a Institute of Virology and AIDS Research, The First Hospital of Jilin University, Changchun, China; b School of Chemistry and Chemical Engineering, Henan Normal University, Xinxiang, China; c Beijing Institute of Radiation Medicine, Beijing, China; Loyola University Chicago

**Keywords:** enteroviruses, FNC, inhibition, viral RNA polymerase, mouse model

## Abstract

Human enteroviruses (EVs), including coxsackieviruses, the numbered enteroviruses, and echoviruses, cause a wide range of diseases, such as hand, foot, and mouth disease (HFMD), encephalitis, myocarditis, acute flaccid myelitis (AFM), pneumonia, and bronchiolitis. Therefore, broad-spectrum anti-EV drugs are urgently needed to treat EV infection. Here, we demonstrate that FNC (2'-deoxy-2'-β-fluoro-4'-azidocytidine), a small nucleoside analog inhibitor that has been demonstrated to be a potent inhibitor of HIV and entered into a clinical phase II trial in China, potently inhibits the viral replication of a multitude of EVs, including enterovirus 71 (EV71), coxsackievirus A16 (CA16), CA6, EVD68, and coxsackievirus B3 (CVB3), at the nanomolar level. The antiviral mechanism of FNC involves mainly positive- and negative-strand RNA synthesis inhibition by targeting and competitively inhibiting the activity of EV71 viral RNA-dependent RNA polymerase (3D^pol^), as demonstrated through quantitative real-time reverse transcription-PCR (RT-qPCR), *in vitro* 3D^pol^ activity, and isothermal titration calorimetry (ITC) experiments. We further demonstrated that FNC treatment every 2 days with 1 mg/kg of body weight in EV71 and CA16 infection neonatal mouse models successfully protected mice from lethal challenge with EV71 and CA16 viruses and reduced the viral load in various tissues. These findings provide important information for the clinical development of FNC as a broad-spectrum inhibitor of human EV pathogens.

**IMPORTANCE** Human enterovirus (EV) pathogens cause various contagious diseases such as hand, foot, and mouth disease, encephalitis, myocarditis, acute flaccid myelitis, pneumonia, and bronchiolitis, which have become serious health threats. However, except for the EV71 vaccine on the market, there are no effective strategies to prevent and treat other EV pathogen infections. Therefore, broad-spectrum anti-EV drugs are urgently needed. In this study, we demonstrated that FNC, a small nucleoside analog inhibitor that has been demonstrated to be a potent inhibitor of HIV and entered into a clinical phase II trial in China, potently inhibits the viral replication of a multitude of EVs at the nanomolar level. Further investigation revealed that FNC inhibits positive- and negative-strand RNA synthesis of EVs by interacting and interfering with the activity of EV71 viral RNA-dependent RNA polymerase (3D^pol^). Our findings demonstrate for the first time that FNC is an effective broad-spectrum inhibitor for human EV pathogens.

## Abstract (precis)

Human enterovirus (EV) pathogens cause various contagious diseases such as hand, foot, and mouth disease, encephalitis, myocarditis, acute flaccid myelitis, pneumonia, and bronchiolitis, which have become serious health threats. However, except for the EV71 vaccine on the market, there are no effective strategies to prevent and treat other EV pathogen infections. Therefore, broad-spectrum anti-EV drugs are urgently needed. In this study, we demonstrated that FNC, a small nucleoside analog inhibitor that has been demonstrated to be a potent inhibitor of HIV and entered into a clinical phase II trial in China, potently inhibits the viral replication of a multitude of EVs at the nanomolar level. Further investigation revealed that FNC inhibits positive- and negative-strand RNA synthesis of EVs by interacting and interfering with the activity of EV71 viral RNA-dependent RNA polymerase (3D^pol^). Our findings demonstrate for the first time that FNC is an effective broad-spectrum inhibitor for human EV pathogens.

## INTRODUCTION

The genus Enterovirus of the *Picornaviridae* family is divided into 13 species. Human enteroviruses (EVs) comprise the first four species, enteroviruses A to D, which include many common important pathogens, such as enterovirus 71 (EV71), coxsackievirus A16 (CA16), CA6, coxsackievirus B3 (CVB3), and enterovirus D68 (EVD68). These human pathogens cause various contagious diseases, including hand, foot, and mouth disease (HFMD), encephalitis, myocarditis, acute flaccid myelitis (AFM), pneumonia, bronchiolitis, and so on (1–6). Some EVs have emerged as serious threats to human health, particularly EV71 and CA16, which cause mainly HFMD in children and infants under 5 years, a disease that is usually mild and self-limiting (7, 8). However, they sometimes result in severe complications, such as brainstem encephalitis, aseptic meningitis, acute flaccid paralysis, and even death (9–11). In the past 2 decades, several large outbreaks have occurred in the Asia-Pacific region (12, 13). In recent years, CA6 and CA10 have been reported to be responsible for HFMD (14–17). In China, the annual HFMD incidence has increased from 37.6/100,000 in 2008 to 139.6/100,000 in 2014 (18, 19). EVD68 has been considered a rare pathogen, but it has recently attracted increased attention due to a wide outbreak in North America in 2014 (20). EVD68 replicates in the respiratory tract and causes respiratory illness, including severe bronchiolitis, pneumonia, and AFM (2, 6, 21). Although the China Food and Drug Administration has approved an EV71 vaccine that is on market, due to the limitation that it only prevents EV71-induced HFMD, there are no effective strategies to prevent and treat EV infection. Therefore, broad-spectrum anti-EV drugs need to be urgently developed.

Enterovirus spp. are small nonenveloped viruses that enclose a positive-sense, single-stranded RNA molecule of approximately 7,400 bases. The viral RNA genome not only is the mRNA for viral protein translation but also can be the template for replication by the virus-encoded RNA-dependent RNA polymerase (RdRP), designated 3D^pol^. Intense research for developing anti-EV71 candidates has been conducted from target-based chemical design to compound screening, even repurposing compounds against poliovirus and human rhinoviruses (22). Some EV inhibitors have been investigated and tested in clinical trials (2, 23). Ribavirin is a broad-spectrum antiviral drug that has been used for treating hepatitis C virus infections and severe respiratory syncytial virus infections (24–26) and was found to inhibit EV71 in RD cells, with a 50% effective concentration (EC_50_) of 65 mg/ml (266 mM), and prevent EV71-induced paralysis and death in mice (27). DTriP-22, a nonnucleoside analog, was demonstrated to inhibit EV71 infection by targeting 3D^pol^ (28). Aurintricarboxylic acid could prevent EV71 infection through interference with 3D^pol^ in Vero cells (29). Apigenin and emetine inhibit EV71 by suppressing viral internal ribosome entry site (IRES) activity (30, 31).

Fluoronucleosides can be well phosphorylated by cellular kinases and are good substrates for RNA and DNA polymerases. Among these fluoronucleosides, 2′-deoxy-2′-β-fluoro-4′-azidocytidine, also known as azvudine or FNC, is a novel cytidine analog that is an excellent substrate for deoxycytidine kinase and can be phosphorylated with higher efficiency than deoxycytidine (32). Previous studies have demonstrated that FNC is a potent inhibitor of hepatitis C virus (HCV) (32, 33) and human and duck hepatitis B virus (HBV) replication (34, 35). In addition, FNC inhibits cell proliferation and promotes apoptosis in a number of human cancer cell lines (36, 37). Chang et al. further proved that FNC possessed strong antiviral activity for human immunodeficiency virus (HIV) (38, 39). Based on this discovery, they have applied and been approved to conduct a clinical trial (NCT 04109183; Clinicaltrials.gov) to evaluate the curative effect of FNC on HIV by the China Food and Drug Administration. So far, the clinical trial has proceeded smoothly to the third stage.

To exploit the potential anti-EV activity of FNC, FNC and 7 derivatives were selected to test their inhibitory activities against EV71 and other EVs in this study. The results showed that only FNC exhibited inhibition potency, with an EC_50_ of 1.548 to 52.12 nM, against a variety of enteroviruses, including EV71, CA16, CA6, CVB3, and EVD68, whereas the 50% cytotoxic concentration (CC_50_) was 3.238 μM in rhabdomyosarcoma (RD) cells, suggesting that FNC has good selectivity indices (CC_50_/EC_50_) against EVs. Interestingly, we observed that FNC exhibited no effect on respiratory syncytial virus (RSV) and influenza A virus (IAV). Further investigation revealed that FNC inhibits RNA synthesis by targeting 3D^pol^. Our study discovered for the first time that FNC could be regarded as an effective candidate inhibitor for EV infection, and the ongoing phase II clinical trial of FNC against HIV which suggests that the safety of FNC will promote FNC’s clinical application against EV infection.

## RESULTS

### Inhibitory effect of FNC and its derivatives on EV71 replication.

**FIG 1:**
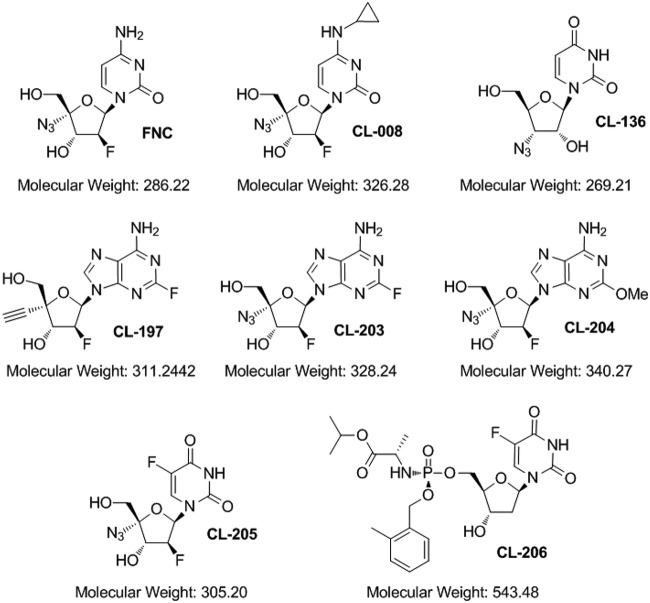
Chemical structures of the eight nucleoside analogs used in this study.

**FIG 2:**
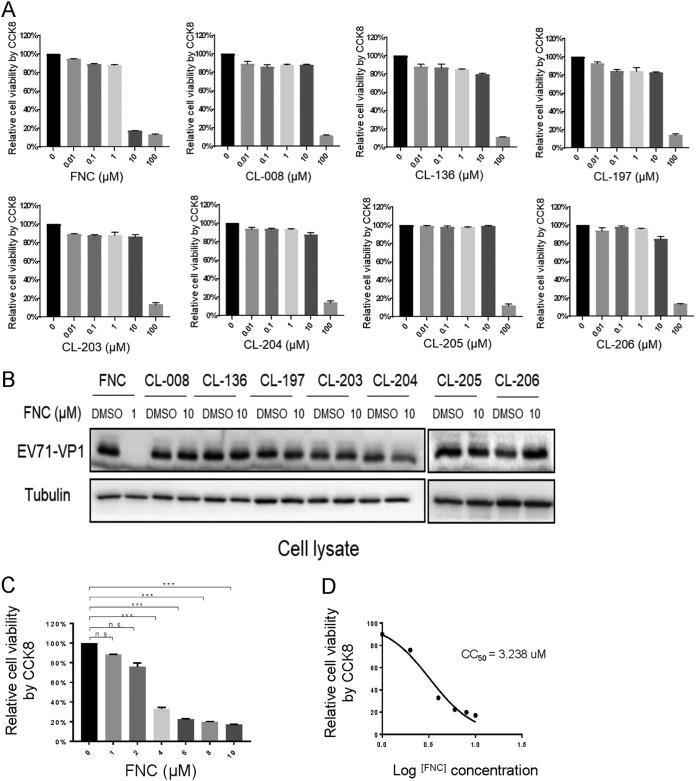
Cytotoxicity and anti-EV71 activity of FNC and its 7 analogs. (A) The dose-dependent cytotoxicity profile of the 8 compounds was detected using a CCK-8 kit in RD cells at 48 h. (B) Anti-EV71 activities of the 8 compounds in the safe-dose range. RD cells were infected with EV71 at an MOI of 0.1 for 4 h, washed twice with DMEM, and supplemented with DMEM containing the indicated concentration of the 8 compounds for another 48 h. The cells were harvested for Western blot (WB) analysis, and tubulin was used as a loading control. (C) Cytotoxicity of FNC in RD cells was detected at 0 to 1 μM at 48 h. (D) The CC_50_ of FNC in RD cells was calculated using GraphPad Prism7.

Previous studies demonstrated that FNC, a nucleoside inhibitor, could significantly inhibit HCV, HIV, and HBV replication by blocking viral DNA or RNA synthesis (32, 34, 35, 38). Here, we investigated whether FNC and its 7 chemical analogs inhibit EV71 infection (Fig. 1). To evaluate the safety of the drugs, we measured the cytotoxic effects of FNC and 7 derivatives on RD cells. RD cells were treated with various concentrations (0.01 to ∼100 μM) of 8 compounds for 48 h. The results of the CCK‑8 assay revealed that FNC did not significantly affect cell viability at a concentration of 1 μM, and the other 7 compounds had no cytotoxic effect at a concentration of 10 μM, indicating that the 8 compounds, particularly FNC, were weakly cytotoxic to RD cells (Fig. 2A). Then, the inhibitory effects of the 8 compounds on EV71 replication were detected in the safe concentration range. RD cells were infected with EV71 for 4 h and then washed and treated with 8 compounds for another 48 h. The expression of intracellular viral VP1 protein detected by Western blotting showed that only FNC possessed obvious anti-EV71 activity at a concentration of 1 μM (Fig. 2B). Next, the cytotoxic effect of FNC on RD cells was measured in a narrow drug concentration range (0 to ∼10 μM) (Fig. 2C), and the CC_50_ of FNC on RD cells was calculated to be 3.238 μM *in vitro* (Fig. 2D). These data showed that FNC was a potent anti-EV71 candidate with relatively low toxicity.

### FNC potently suppresses EV71 replication at the nanomolar level.

**FIG 3:**
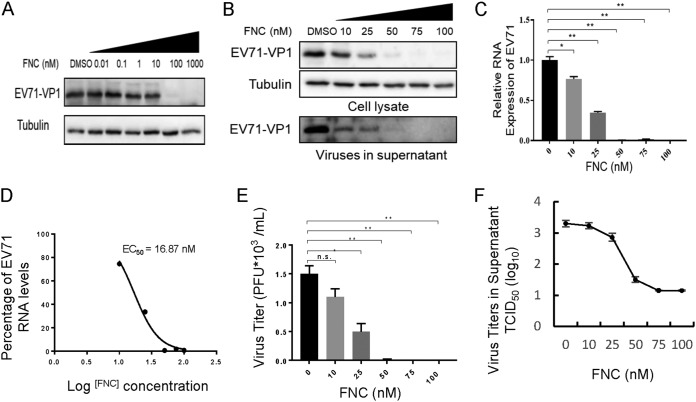
FNC potently inhibits the replication of EV71 in a dose-dependent manner. (A and B) Inhibitory effect of FNC on EV71 replication in the 0.01 to 1,000 nM (A) or 10 to 100 nM (B) dose range. RD cells were infected with EV71 as described in Fig. 2B, and the indicated concentration of FNC was added to RD cells for 48 h. The cells were then harvested for WB analysis. The supernatant from the infected RD was centrifuged and then loaded for WB analysis. Tubulin was used as a loading control. (C) Cellular EV71 RNA levels in panel B were detected by RT-qPCR. GAPDH was used as a control. The EV71 RNA level without FNC treatment was set as 100%. (D) The EC_50_ of FNC was calculated according to panel C using GraphPad Prism7. (E and F) Viral titers in the supernatants from the experiment shown in panel B were determined by the plaque assay (E) and by the cytopathic effect (CPE) method (F). The results shown are the means with SDs from two independent experiments. The asterisks indicate statistically significant differences between groups, as assessed by Student's *t* test (*, *P* ≤ 0.05; **, *P* ≤ 0.01; and ***, *P* ≤ 0.001; ns, not significant).

To further demonstrate the ability of FNC to inhibit EV71 propagation, the expression levels of the EV71 VP1 protein and viral RNA were examined. Western blot analysis showed that the EV71 VP1 protein could not be detected when RD cells were treated with 100 nM FNC for another 48 h postinfection (Fig. 3A). Accordingly, FNC had an obviously dose-dependent inhibitory effect on the propagation of EV71, as demonstrated by detecting EV71 VP1 expression in cells and culture supernatants (Fig. 3B). In addition, quantitative real-time reverse transcription-PCR (RT-qPCR) assays revealed that FNC signiﬁcantly reduced the total RNA level of EV71 in infected RD cells (Fig. 3C). According to the reduced viral RNA level, the EC_50_ of FNC against EV71 was calculated to be 16.87 nM (Fig. 3D). Unsurprisingly, continuously decreasing virus titers in the supernatants of EV71-infected RD cells were observed with increasing doses of FNC by a plaque assay (Fig. 3E) and cytopathic effect (CPE)-based 50% tissue culture infective dose (TCID_50_) assay (Fig. 3F). These results suggested that FNC is a potent inhibitor of EV71.

### FNC exhibits potent antiviral activity against a broad range of EVs but not respiratory syncytial virus or influenza A virus.

**FIG 4:**
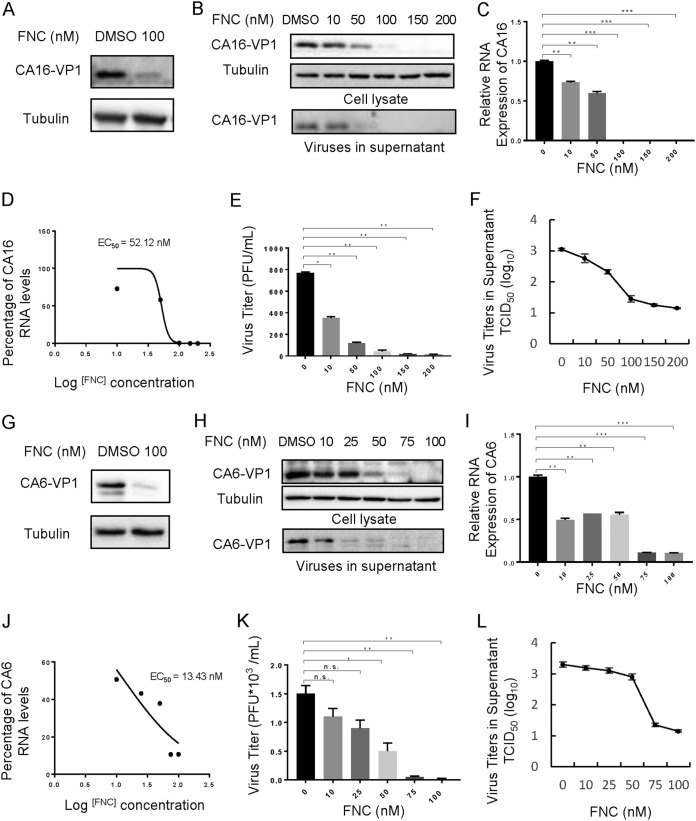
FNC potently inhibits the replication of CA16 and CA6 in a dose-dependent manner. (A and G) A 100 nM dose of FNC inhibited CA16 (A) and CA6 (G) viral replication in RD cells. (B) The inhibitory effect of FNC on CA16 replication in the 10 to 200 nM dose range. The CA16 VP1 protein in cell lysate or culture supernatant was detected by WB analysis, and tubulin was used as a loading control. (C) CA16 RNA levels in the cells from the experiments shown in panel B were detected by RT-qPCR. GAPDH was used as a control. The CA16 RNA level without FNC treatment was set as 100%. (D) The EC_50_ of FNC was calculated according to panel C using GraphPad Prism7. (E and F) The viral titers in the supernatants from the experiment shown in panel B were determined by the plaque assay (E) and by the cytopathic effect (CPE) method (F). (H) The inhibitory effect of FNC on CA6 replication in the 10 to 100 nM dose range. The CA6 VP1 protein in cell lysate or culture supernatant was detected by WB analysis, and tubulin was used as a loading control. (I) CA6 RNA levels in the cells from the experiments shown in panel H were detected by RT-qPCR. GAPDH was used as a control. The CA6 RNA level without FNC treatment was set as 100%. (J) The EC_50_ of FNC was calculated according to panel I using GraphPad Prism7. Viral titers in the supernatants from the experiment shown in panel H were determined by the plaque assay (K) and by the CPE method (L). The results are shown the means with SDs from two independent experiments. The asterisks indicate statistically significant differences between groups, as assessed by Student's *t* test (*, *P* ≤ 0.05; **, *P* ≤ 0.01; and ***, *P* ≤ 0.001; ns, not significant).

**FIG 5:**
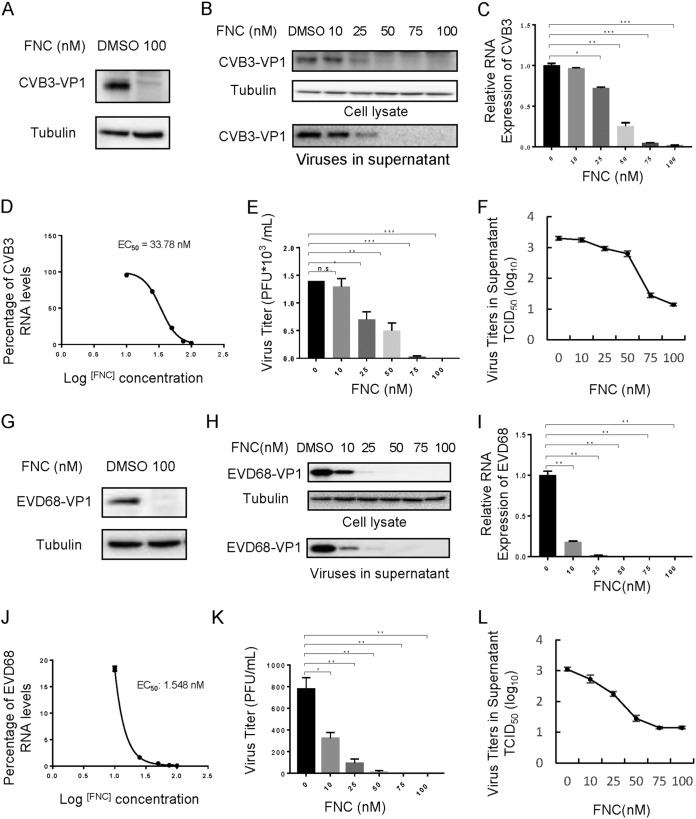
FNC potently inhibits the replication of CVB3 and EVD68 in a dose-dependent manner. (A and G) A 100 nM dose of FNC inhibited CVB3 (A) and EVD68 (G) viral replication in RD cells. (B) The inhibitory effect of FNC on CVB3 replication is in the 10 to 100 nM dose range. The CVB3 VP1 protein in cell lysate or the culture supernatant was detected by WB analysis, and tubulin was used as a loading control. (C) CVB3 RNA levels in the cells from the experiments shown in panel B were detected by RT-qPCR. GAPDH was used as a control. The CVB3 RNA level without FNC treatment was set as 100%. (D) The EC_50_ of FNC was calculated according to panel C using GraphPad Prism7. (E and F) Viral titers in the supernatants from the experiment shown in panel B were determined by the plaque assay (E) and by the cytopathic effect (CPE) method (F). (H) The inhibitory effect of FNC on EVD68 replication in the 10 to 100 nM dose range. The EVD68 VP1 protein in cell lysate or culture supernatant was detected by WB analysis, and tubulin was used as a loading control. (I) EVD68 RNA levels in the cells from the experiments shown in panel H were detected by RT-qPCR. GAPDH was used as a control. The EVD68 RNA level without FNC treatment was set as 100%. (J) The EC_50_ of FNC was calculated according to panel I using GraphPad Prism7. (K and L) Viral titers in the supernatants from the experiment shown in panel H were determined by the plaque assay (K) and by the CPE method (L). The results shown are the means with SDs from two independent experiments. The asterisks indicate statistically significant differences between groups, as assessed by Student's *t* test (*, *P* ≤ 0.05; **, *P* ≤ 0.01; and ***, *P* ≤ 0.001; ns, not significant).

**FIG 6:**
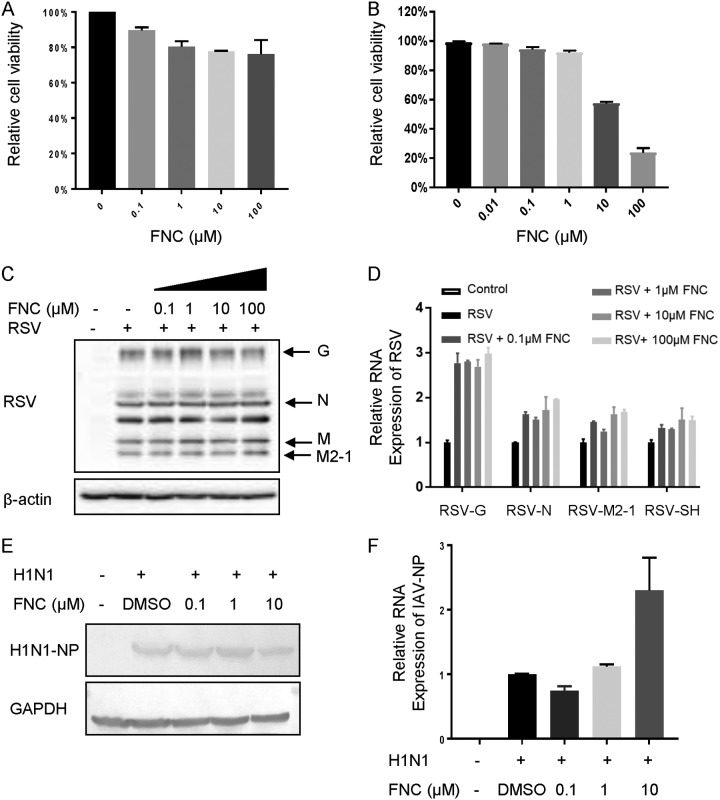
FNC has no inhibitory effect on RSV and IAV. (A and B) Cytotoxicity of FNC in HEK293T (A) or MDCK (B) cells was detected using a CCK-8 kit at 48 h. (C) HEK293T cells were infected with RSV at an MOI of 0.5 for 4 h, washed twice with DMEM, and supplemented with DMEM containing the indicated concentration of FNC for another 48 h. The cells were harvested. Representative structural proteins G, N, M, and M2-1 were detected by WB analysis, and tubulin was used as a loading control. (D) RSV RNA levels in cells were detected by RT-qPCR. GAPDH was used as a control. The RSV RNA level without FNC treatment was set as 100%. (E) MDCK cells were infected with IAV H1N1 at an MOI of 0.1 for 4 h, washed twice with DMEM, and supplemented with DMEM containing the indicated concentration of FNC for another 48 h. The cells were harvested for WB analysis, and tubulin was used as a loading control. (F) IAV RNA levels in cells were detected by RT-qPCR. GAPDH was used as a control. The IAV RNA level without FNC treatment was set as 100%.

There is a great need for broad-spectrum anti-EV drugs. Therefore, we investigated the effect of FNC on other EVs, including CA16, CA6, CVB3, and EVD68. Viral VP1 protein and RNA levels, as well as viral titers in the supernatant of infected RD cells, were detected postinfection. As shown in Fig. 4 and 5, FNC potently inhibited VP1 protein expression and total viral RNA levels in RD cells infected with the tested EVs. The EC_50_ values of FNC for CA16, CA6, CVB3, and EVD68 were 52.12 nM, 13.43 nM, 33.78 nM, and 1.548 nM (Fig. 4D and J and 5D and J), respectively, according to decreased RNA levels (Fig. 4C and I and 5C and 5I). Virions in the supernatant and viral titers were also suppressed by FNC in a dose-dependent manner, which was consistent with the reduction in total RNA levels (Fig. 4B, E, F, H, K, and L and 5B, E, F, H, K, and L). To investigate the specificity of FNC activity, the antiviral activities of FNC against respiratory syncytial virus (RSV) and influenza A(H1N1) virus were further investigated. First, we observed that the safe doses of FNC on HEK293T cells and MDCK cells were 100 μM and 10 μM, respectively, by treatment with various concentrations (0.01 to ∼100 μM) of FNC for 48 h (Fig. 6A and B). Next, viral proteins and RNA levels of RSV and H1N1 were detected by Western blotting and RT-qPCR under safe drug concentrations (Fig. 6C to F). We observed that FNC had no inhibitory effect on RSV and H1N1 replication in HEK293T and MDCK cells, indicating that FNC inhibits EVs but not RSV or A(H1N1) influenza virus.

### FNC inhibits EV RNA synthesis by binding to RNA polymerase 3D^pol^.

**FIG 7:**
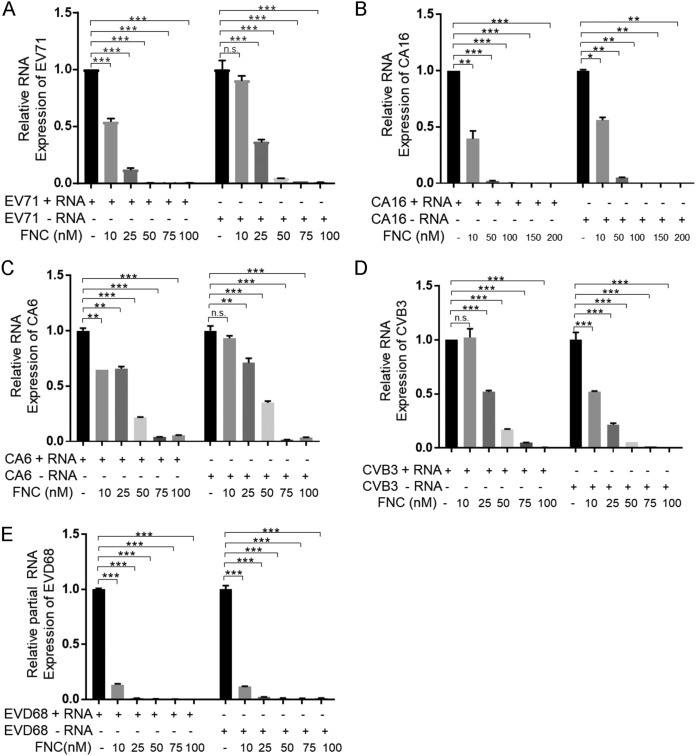
FNC inhibits the transcription of EVs through interacting with 3D^pol^. (A to E) FNC inhibited both positive- and negative-strand RNA production of EV71 (A), CA16 (B), CA6 (C), CVB3 (D), and EVD68 (E). RD cells were infected with the indicated EVs, treated with the indicated concentration of FNC for 48 h, and harvested for RNA extraction and RT-qPCR. GAPDH was used to normalize the cells. The asterisks indicate statistically significant differences between groups, as assessed by Student's *t* test (*, *P* ≤ 0.05; **, *P* ≤ 0.01; and ***, *P* ≤ 0.001; ns, not significant).

**FIG 8:**
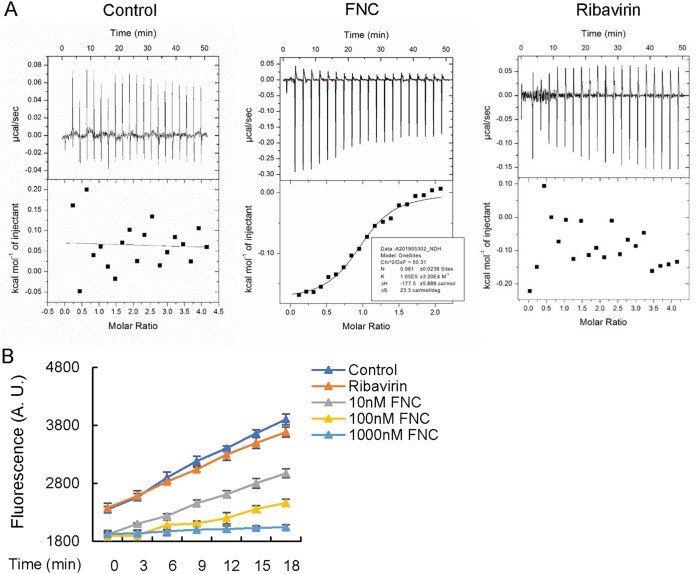
FNC inhibits the activity of 3D^pol^ by direct interaction. (A) ITC binding curves for EV71 3D^pol^ protein and negative control (buffer) or FNC or ribavirin at 25°C showed that FNC binds to EV71 3D^pol^ with a *K_d_* of 6 × 10^−6^ mol/liter. The titrant of 1.0 mM FNC or 3 mM ribavirin was mixed with 100 μM EV71 3D^pol^ protein solution. Curves in the bottom graphs show the fitting of data to a one-set model by the nonlinear Levenberg-Marquardt fitting algorithm. (B) Fluorescence-based polymerization assay using EV71 3D^pol^. Experiments were carried out as described in Materials and Methods. The results shown are the means with SDs from three independent experiments. A.U., arbitrary units.

It has been proven that FNC can efficiently inhibit HCV replication by binding to HCV RdRp NS5B, causing chain termination of RNA synthesis (32). Therefore, we detected whether FNC suppressed EV replication by affecting RdRp-mediated viral RNA synthesis. We observed that FNC inhibited both positive- and negative-strand EV RNA synthesis, including that for EV71, CA16, CV6, CVB3, and EVD68, by RT-qPCR detecting positive- or negative-strand EV RNA after incubation with the tested concentrations of FNC (Fig. 7A to E). For EV71, CA16, and CA6, FNC showed stronger inhibition against positive-strand than negative-strand RNA synthesis. However, the inhibitory effect of FNC on CBV3 and EVD68 was the opposite. EVs encode RNA-dependent RNA polymerase, denoted 3D^pol^, which is essential for viral genome replication. Therefore, we speculated that FNC might be incorporated into nascent RNA by binding to 3D^pol^ and causing chain termination. We next expressed and purified EV71 3D^pol^ in Escherichia coli and detected the interaction between FNC and EV71 3D^pol^ by isothermal titration calorimetry (ITC) (Fig. 8A). Compared with the negative control (buffer), we found that FNC directly interacted with EV71 3D^pol^ with a *K_d_* (dissociation constant) of 6 × 10^−6^ mol/liter. As a broad-spectrum antiviral drug, ribavirin is currently used to treat several RNA virus infections clinically. A previous study showed that ribavirin effectively reduced EV71 viral yields (EC_50_, 65 μg/ml [266 mM]) by detection using a virus-induced CPE assay (27). However, we failed to observe the interaction between ribavirin and EV71 3D^pol^ by an ITC assay, suggesting that the inhibitory effect of ribavirin on EV71 is not related to a direct interaction with EV71 3D^pol^, which is consistent with previous report by Lohmann et al. showing that ribavirin inhibition on HCV is not related to a direct inhibition of the RdRp NS5B of HCV (40). Actually, ribavirin is a broad-spectrum antiviral agent and nonselective nucleoside analog, which is different from FNC, with a higher EC_50_ value (266 mM) than the EC_50_ value of FNC (24.73 nM) for EV71 inhibition. Furthermore, a fluorescence-based 3D^pol^ activity assay indicated that FNC inhibited the RNA synthesis activity of EV71 3D^pol^ in a dose-dependent manner (Fig. 8B). It suggested that FNC could more efficiently restrain EV RNA synthesis, especially positive-strand RNA synthesis, than could ribavirin by binding to EV 3D^pol^.

### FNC protects neonatal mice from lethal EV71 or CA16 challenge.

**FIG 9:**
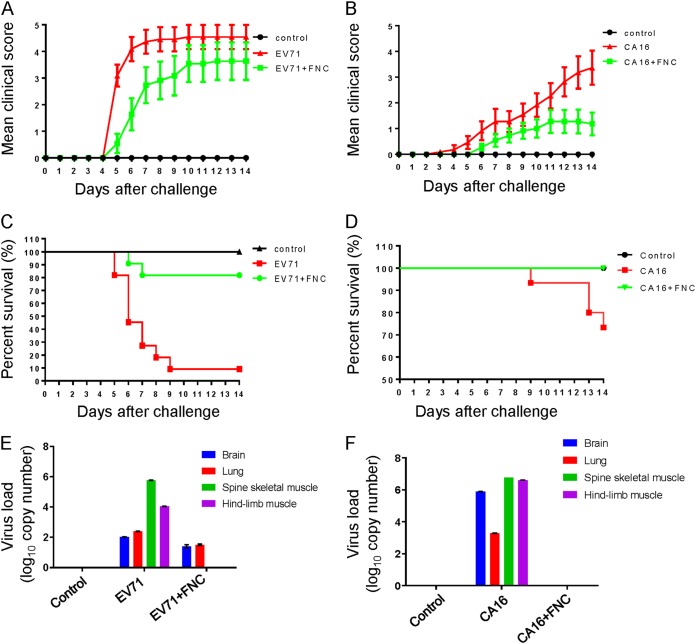
FNC protects neonatal mice from EV71 and CA16 lethal challenges. (A to F) One-day-old ICR mice (*n* = 8 to 10) were inoculated with 10 μl of EV71 CC063 at 10^5.5^ CCID_50_ ml^−1^ (A, C, and E) or CA16 CC045 at 10^2.7^ CCID_50_ ml^−1^ (B, D, and F) and at 1 h postchallenge injected intraperitoneally with FNC at days 1, 3, 6, 9, and 12. Clinical scores and survival rates were monitored for 15 days postchallenge. Various grades of clinical disease were identified as follows: 0, healthy; 1, lethargy and inactivity; 2, wasting; 3, limb-tremor weakness; 4, hind-limb paralysis; and 5, moribund or dead. (E and F) Viral loads of EV71 (E) and CA16 (F) in samples of the brain, lung, spine skeletal muscle, and hind-limb muscle tissues of infected mice were assessed by RT-qPCR at day 8. The results represent the mean virus loads (log_10_ copies/mg tissue) ± SDs (three mice per group, repeated three times).

In a previous study, we established a lethal EV71 and CA16 infection neonatal mouse model (41, 42). Here, we investigated whether FNC protects neonatal mice against lethal challenge by EV71 and CA16 viruses. We intracerebrally injected the Changchun-circulating EV71 CC063 (each at 10^5.5^ 50% cell culture infective dose [CCID_50_] ml^−1^) and CA16 CC045 (each at 10^2.7^ CCID_50_ ml^−1^) into 1-day-old mice, followed by FNC (infected and treated groups) or dimethyl sulfoxide (DMSO) treatment (infected and untreated groups) (Fig. 9). For the FNC-treated group, the mice were treated with FNC (1 mg/kg of body weight) on days 1, 3, 6, 9, and 12, and for the untreated group, the mice were treated with the same volume of DMSO at the same time. The clinical scores and survival rates of infected mice were monitored for 15 days post-virus challenge. The results showed that mice infected with EV71 CC063 and CA16 CC045 became sick on day 5 and day 8 postinfection and presented a gradual aggravation tropism, with a clinical score of grade 4 on day 6 for EV71 CC063 and a clinical score of grade 3 on day 12 for CA16 CC045 (Fig. 9A and B). Compared to the FNC-treated mice, the DMSO-treated mice exhibited observable illness symptoms, such as single-limb paralysis, which occurred approximately 2 days earlier. At day 9 post-EV71 challenge, the status of the DMSO-treated mice deteriorated, reaching a peak of death or symptoms such as four-limb paralysis, whereas mice with the FNC intervention presented fewer deaths or two-limb paralysis. Importantly, the mortality due to EV71 and CA16 challenge reached 90% and 30%, respectively, while FNC treatment greatly reduced the mortality to 20% and 0% (Fig. 9C and D). FNC treatment significantly improved clinical manifestations and survival rates, indicating that FNC effectively protected against EV71 and CA16 challenge *in vivo*.

**FIG 10:**
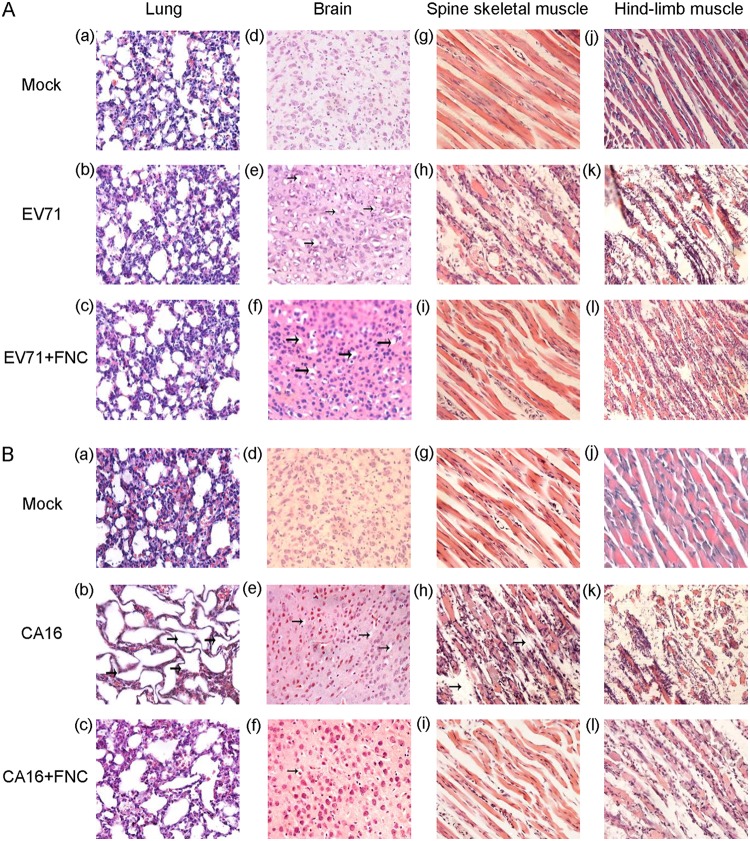
Pathology analysis of EV71- or CA16-infected neonatal mice. One-day-old ICR mice were intracerebrally inoculated with EV71 CC063 (10^5.5^ CCID_50_/ml), CA16 CC045 (10^2.7^ CCID_50_/ml), or DMEM (mock control). At 1 h postchallenge, FNC or DMSO was injected intraperitoneally, as described in Materials and Methods. (A and B) Representative images from the lung (a to c), brain (d to f), spine skeletal muscle (g to i), and hind-limb muscle (j to l) postinfection are shown for EV71-infected (A) or CA16-infected (B) mice. The images for noninfected mice were used as a control (a, d, g, and j in panels A and B). Magnification, ×400.

To further confirm the antiviral activity of FNC *in vivo*, viral RNA was examined by RT-qPCR in some tissues, such as the brain, lung, spine skeletal muscle, and hind-limb muscle, where EV71 and CA16 tend to replicate (41, 42). As shown in Fig. 9E, FNC treatment completely decreased the EV71 viral load in the spine skeletal muscle and hind-limb muscle and moderately reduced viral loads in the brain and lung compared to those in the DMSO treatment group (*P* < 0.05). In addition, FNC treatment completely decreased the CA16 viral load in all detected tissues compared to those in the DMSO treatment group (*P* < 0.05) (Fig. 9F). Meanwhile, tissues (mock, EV71-infect, or CA16-infected and DMSO treatment and EV71- or CA16-infected and FNC treatment) of mouse lung, brain, spine skeletal muscle, and hind-limb muscle were sampled and histologically examined by hematoxylin and eosin (H&E) staining (Fig. 10). As shown in Fig. 10Aa, d, g, and j and Ba, d, g, and j, no obvious histological changes were observed in the mock-infected mice. In contrast, EV71 and CA16 infection both resulted in severe necrosis, including significant muscle bundle fracture and dissolution of muscle fiber cells in the spinal skeletal muscle and the hind-limb muscle of EV71- or CA16-infected mice (Fig. 10Ah and k and Bh and k). Also, CA16 but not EV71 infection caused obvious lung lesions, including severely enlarged alveoli (Fig. 10Ab and Bb). Patchy vacuoles in the brain were observed (Fig. 10Ae and Be). These results indicated definite damages on mice tissue caused by EV71 and CA16 infection. However, FNC treatment significantly reduced the damage to mouse tissues of EV71-infected spinal skeletal muscle and hind-limb muscle and CA16-infected brain, spinal skeletal muscle, and hind-limb muscle (Fig. 10Ai and l and Bf, i, and l). Alleviated lesions were found in the EV71-infected brain and CA16-infected lung (Fig. 10Af and Bc). These results are basically consistent with the viral loads of infected mouse tissues in Fig. 9E and F. Together, these results suggest that severe tissue damage might be the major cause of death from EV71 infection and that FNC can effectively alleviate these effects (43).

## DISCUSSION

HFMD, encephalitis, aseptic meningitis, myocarditis, acute flaccid myelitis, pneumonia, bronchiolitis, and so on caused by multiple human EVs, including EV71, CA16, CA6, CA10, CVB3, and EVD68, have been severe public health problems for the last 2 decades. At present, there are no broad-spectrum antiviral or prophylactic agents available for EV infections. FNC (2′-deoxy-2′-β-fluoro-4′-azidocytidine), also known as azvudine, is a novel cytidine analog that is an excellent substrate for deoxycytidine kinase and can be phosphorylated with efficiencies up to 3-fold higher than for deoxycytidine (32). Previous studies have demonstrated that FNC is a potent inhibitor of HIV, HCV, and HBV replication (32, 34, 35, 38). In this study, we evaluated the cytotoxicity and antiviral activities of FNC as well as its analogs against multiple EVs, including EV71, CA16, CA6, EVD68, and CVB3, and observed that FNC showed relatively low cytotoxicity toward RD cells (safe dose of 1 μM), HEK293T cells (safe dose of 100 μM), and MDCK cells (safe dose of 10 μM) (Fig. 2A and 6A and B). We found that FNC but not its 7 analogs efficiently suppressed these EVs in an *in vitro* cell model by detecting viral VP1 protein expression, viral RNA levels, and viral titers. The EC_50_ is 1.548 to ∼52.12 nM (Fig. 3 to 5). Taken together, these results demonstrate that FNC has good selectivity indices (CC_50_/EC_50_) against EVs. Interestingly, we observed that FNC had no inhibitory effect on RSV and IAV, indicating the specificity of FNC against EVs (Fig. 6). This phenomenon had been reported by previous studies showing that R1479 (4′-azidocytidine) showed high selectivity for the inhibition of HCV replication, while R1479-TP did not inhibit the related RdRp of influenza virus. The increased antiviral selectivity might due to the introduction of a 4′-azido group to compounds (32, 44, 45). Moreover, RSV and IAV both belong to single-minus-strand RNA viruses, which are different from single-plus-strand EVs. RNA-dependent RNA polymerase from various RNA viruses, for example, IAV 3D^pol^ composed of three subunits, is totally different from EV’s 3D^pol^. Therefore, FNC has different effects on various RNA viruses, even enhancing IAV and RSV replication to a small degree. Thus, we should be careful to use FNC for the treatment of EVs in the future, especially when EVs and RSV or IAV are present as a coinfection.

Generally, targeting viral proteins or essential host factors required for virus replication is the major strategy for antiviral drug development. Drugs targeting viral proteins have limited side effects (23). Nucleoside analogs such as ribavirin and 2′-C-methylcytidine have been studied most extensively as RNA virus infection inhibitors (24, 27). Previous studies revealed that the main antiviral mechanism of FNC against HCV functions by preventing viral RNA replication events by targeting NS5B, the RdRp encoded by HCV (33). Since the EV RNA genome is replicated by its RdRp, known as the 3D^pol^ protein, targeting the 3D polymerase could be a potent strategy for specifically inhibiting EV replication. Hence, we speculated that FNC might inhibit EVs by blocking viral RNA synthesis. In this study, by detecting positive- or negative-strand EV RNA postincubation with the tested concentrations of FNC, we observed that FNC inhibited the replication of both positive- and negative-strand EV RNA synthesis, including that of EV71, CA16, CV6, CVB3, and EVD68 (Fig. 7). Moreover, an ITC assay further confirmed the interaction of FNC and EV71 3D^pol^ (Fig. 8A). It is well known that FNC is an excellent substrate for deoxycytidine kinase and can be phosphorylated more efficiently than deoxycytidine (33). Here, we used FNC but not its phosphorylated form to titrate EV71 3D^pol^ binding, and we did observe the interaction between them (*K_d_* = 6 × 10^−6^ mol/liter). We believe that phosphorylated FNC should possess stronger affinity to 3D^pol^ than does FNC in cells. *In vitro* 3D^pol^ activity assay also showed that FNC inhibited the RNA synthesis activity of EV71 3D^pol^ in a dose-dependent manner (Fig. 8B). This phenomenon is similar to the mechanism of the inhibition of HCV replication by RO-0622, which is efficiently incorporated into nascent RNA by RdRp NS5B of HCV and causes chain termination (32). For HIV inhibition, FNC with a 3′-OH group also causes the chain termination of proviral DNA biosynthesis (38).

Based on the strong anti-EV replication ability that FNC exhibited *in vitro*, we further evaluated the protective effect of FNC in an EV71- or CA16-challenged neonatal mouse model by employing clinically isolated strains from hospitalized HFMD patients in the northeastern region of China who had severe or mild clinical symptoms (41, 42). As reported, these viruses generated many symptoms in neonatal mice, such as lethargy, hind-limb paralysis, and severe lesions in the lung tissue, including pulmonary edema and hemorrhage. We determined that FNC effectively alleviated the symptoms caused by EV71 and CA16 and reduced the lethal rates of neonatal mice (Fig. 9 and 10). An RT-qPCR assay revealed that FNC decreased the viral loads of EV71 and CA16 in the muscle, brain, and lung tissues compared to those in those tissues of nontreated neonatal mice (Fig. 9). In particular, these clinically isolated strains were demonstrated to be recombinant strains in our previous studies (46, 47), which indicates common circulating strains, suggesting that FNC is a potent candidate against EVs.

In summary, with a combination of *in vitro* and *in vivo* assays, we identified that FNC acts as an effective antiviral compound for various EVs by targeting the function of 3D^pol^. FNC competitively inhibited EV71 infection with an EC_50_ of 16.87 nM and bound EV71 3D^pol^ with a *K_d_* of 6 × 10^−6^ mol/liter. Additionally, FNC had potency against viral replication for extensive virus lineages, including CA16, CA6, CVB3, and EVD68. Moreover, the ongoing phase II clinical trial of FNC against HIV, suggesting that FNC is safe for treatments, will promote its clinical application against EV infection. Therefore, FNC represents a new fluoronucleoside small-molecule inhibitor for further development of antiviral therapy against EVs or other picornaviruses (48–50).

## MATERIALS AND METHODS

### Cells and viruses.

Human rhabdomyosarcoma (RD) cells (ATCC CCL-136), HEK293T cells (ATCC CRL-11268), and MDCK cells (ATCC CRL-2935) were purchased from the American Type Culture Collection (USA) and cultured in Dulbecco’s modiﬁed Eagle’s medium (DMEM; Gibco, USA) with 10% fetal bovine serum (FBS; Gibco). The cell culture plates were incubated in a humidiﬁed incubator containing 5% CO_2_ at 37°C.

EV71 CC063, CA16 CC045, and CA6 (changchun046/CHN/2013) were isolated and preserved by our lab. CA16 (shzh05-1), EVD68 (US/KY/14-18953), and CVB3 (GenBank accession no. JX312064.1) have been described previously (51). Virus particles were ampliﬁed using RD cells, and the virus titer was determined using a plaque reduction assay with RD cells, according to the Reed-Muench formula.

### Compounds.

Eight compounds were synthesized by Chang’s lab (34–39) and dissolved in dimethyl sulfoxide (DMSO) for *in vitro* and *in vivo* studies. FNC and ribavirin were dissolved in buffer (pH 7.5, 25 mM HEPES, 100 mM NaCl) for the ITC experiment.

### Cytotoxicity assays.

The cytotoxicities of the 8 compounds against RD cells, HEK293T cells, and MDCK cells were determined using a cell viability assay in 96-well plates with cells cultured to 80 to 90% conﬂuence (approximately 5 × 10^4^ cells/well). The cells were treated with various concentrations of compounds dissolved in DMSO for 48 h, and then cell viability was assayed using a Cell Counting kit‑8 (CCK-8; Beyotime, Shanghai, China). In brief, 10 μl of the CCK‑8 solution was added, and the cells were incubated for an additional 2 h. Medium without cells was used as a blank control. Absorbance at a wavelength of 450 nm was measured using a microplate reader. Cell viability was determined as a percentage of that of the control. All cell proliferation assays were performed in triplicate and repeated in three independent experiments.

### Antiviral activity *in vitro*.

RD cells, HEK293T cells, and MDCK cells were plated into 24-well culture plates and incubated for 12 h. The medium was then removed, and cells were infected with the designated virus (multiplicity of infection [MOI], 0.1). After virus absorption for 2 h, the medium was aspirated from the wells to remove unabsorbed virus, and the cells were washed three times with serum-free DMEM and treated with different compound concentrations to test for antiviral activity. Finally, virus-infected cells and culture supernatants were collected 48 h after infection for Western blotting, RT-qPCR, and virus titer detection.

### Quantitative real-time reverse transcription-PCR.

At 48 h postinfection, total RNA from the samples was extracted using the TRIzol reagent (Invitrogen, Carlsbad, CA). Each sample was then treated with DNase (Promega) before being quantitatively assayed. Then, the RNA was reverse transcribed using a Transcriptor cDNA synthesis kit 1 (catalog no. 4896866001; Roche, Basel, Switzerland). Quantitative real-time reverse transcription-PCR (RT-qPCR) was carried out on an Mx3005P instrument (Agilent Technologies, Stratagene, USA) by using a master mix (SYBR green) kit (Bio-Rad), and the primers used are listed in Table 1. The RT-qPCR assay was carried out in a 20-μl volume consisting of 10 μl of a 2× SYBR green mix solution, 1 μl of 5 μM each oligonucleotide primer, and 2 μl of the cDNA template. The target fragment amplification was carried out as follows: 95°C for 5 min, followed by 40 cycles of 95°C for 30 s, 60°C for 30 s, and 72°C for 30 s. The threshold cycle (*C_T_*) value of each sample was determined, and the relative mRNA level was normalized to the glyceraldehyde-3-phosphate dehydrogenase (GAPDH) mRNA value.

**TABLE 1:** Primers used for RT-qPCR in this study

Primer name	Primer direction	Sequence (5′–3′)
EV71-RT-F	Forward	CTTTGTGCGCCTGTTTTATAC
EV71-RT-R	Reverse	GGAAACAGAAGTGCTTGATCA
CA16-RT-F1	Forward	CATGCAGCGCTTGTGCTT
CA16-RT-F2	Forward	CATGCAACGACTGTGCTTTC
CA16-RT-R1	Reverse	CACACAATTCCCCCGTCTTACT
CA16-RT-R2	Reverse	CATAATTCGCCCGTTTTGCT
CA6-RT-F	Forward	AATGAGGCGAGTGTGGAAC
CA6-RT-R	Reverse	AGGTTGGACACAAAAGTGAACT
CVB3-RT-F	Forward	GAATGCGGCTAATCCTAACTGC
CVB3-RT-R	Reverse	GCTCTATTAGTCACCGGATGGC
EVD68-RT-F	Forward	CAGTCACAGCCACACTAGC
EVD68-RT-R	Reverse	CAATCTAAACCCCTGAGAGC
Negative-sense-RT-F	Forward	TTAAAACAGCCTGTGGGTTG
Positive-sense-RT-R	Reverse	Oligo d(T)
RSV-G-RT-F	Forward	ACCTGCTGGGCTATCTGC
RSV-G-RT-R	Reverse	TTGGTTGTCTTGAGGGTTG
RSV-N-RT-F	Forward	TGCAGGGCAAGTGATGTTAC
RSV-N-RT-R	Reverse	TTCCATTTCTGCTTGCACAC
RSV-M2-1-RT-F	Forward	TGGCCACCCCATGCACTG
RSV-M2-1-RT-R	Reverse	TCCAACTCTGCAGCTCCAC
RSV-SH-RT-F	Forward	ACATGATCACAACAATAATCTC
RSV-SH-RT-R	Reverse	AAAGGTTTTGTTATGGAATACG
H1N1-NP-RT-F	Forward	TGCTTCAAAACAGCCAAGTG
H1N1-NP-RT-R	Reverse	GATGCCCTCTGTTGATTGGT
GAPDH-RT-F	Forward	TGCACCACCAACTGCTTAGC
GAPDH-RT-R	Reverse	GGCATGGACTGTGGTCATGAG

### SDS-PAGE, Western blot analysis, and antibodies.

Cells from virus-infected 24-well plate samples with appropriate compound treatments were harvested at 12 h postinfection. Samples of virus-infected and drug-treated RD cells, HEK293T cells, and MDCK cells were treated with 1× loading buffer (0.08 M Tris [pH 6.8] with 2.0% SDS, 10% glycerol, 0.1 M dithiothreitol, and 0.2% bromophenol blue) and boiled at 100°C for 30 min. Then, the samples were cleared by centrifugation at 12,000 × *g* and 4°C for 10 min. The total cell extracts were subjected to SDS-PAGE and transferred onto polyvinylidene fluoride (catalog no. BSP0161; Pall) or nitrocellulose (catalog no. 10600001; GE) membranes. After blocking with 5% nonfat dry milk in Tris-buffered saline with Tween 20 (TBST) for 1 h at room temperature (RT), the membranes were incubated with the indicated primary antibodies at 4°C overnight and then with the corresponding horseradish peroxidase (HRP)-conjugated antibody (Millipore) or alkaline phosphatase (AP)-conjugated secondary antibody (Sigma) for 1 h. After three washes with TBST, the proteins were developed using a hypersensitive electrochemiluminescence (ECL) detection kit (catalog no. B500023; Proteintech) or nitro blue tetrazolium (NBT) and 5-bromo-4-chloro-3-indolyl phosphate (BCIP; Sigma).

The following antibodies were used in this study: polyclonal antibodies (pAbs) against EV71 and CA16 were obtained from rabbits immunized with EV71 (diluted 1:200) and CA16 (diluted 1:500) whole viruses in our laboratory, respectively. A pAb against CVB3 was obtained from a mouse immunized with CVB3 (diluted 1:100) whole viruses in our laboratory. A polyclonal rabbit anti-CA6 VP1 antibody (GTX132346, diluted 1:1,000; GeneTex), polyclonal rabbit anti-EVD68 VP1 antibody (GTX132313, diluted 1:500; GeneTex), polyclonal goat anti-RSV antibody (AB1128, diluted 1:250; Millipore), polyclonal rabbit anti-NP antibody (PA5-32242, diluted 1:2,500; Thermo Fisher), anti-tubulin monoclonal antibody (MAb; ab11323, diluted 1:1,000; Abcam, Cambridge, UK), anti-β-actin antibody (A00702-100, diluted 1:2,000; GenScript), anti-GAPDH mouse monoclonal antibody (G8795, diluted 1:1,000; Sigma), goat anti-rabbit peroxidase-conjugated IgG antibody (AP132P, diluted 1:10,000; Millipore), goat anti-mouse peroxidase-conjugated IgG antibody (AP124P, diluted 1:10,000; Millipore), goat anti-mouse IgG (H+L) highly cross-adsorbed secondary antibody (115-055-062, diluted 1:1,000; Jackson ImmunoResearch), and goat anti-rabbit IgG (H+L) highly cross-adsorbed secondary antibody (115-055-003, diluted 1:1,000; Jackson ImmunoResearch) were purchased.

### Plaque assay.

For the *in vitro* experiment, samples were collected (including supernatants and cells) and were prepared for titrating viral titers by undergoing freeze-thaw cycles twice and then centrifuged at 1,000 × *g* and 4°C for 20 min to obtain supernatants. Subsequently, supernatants were passed through a 0.22-μm filter, and a plaque assay was performed. First, RD cells (1.5 × 10^5^ cells/well) were seeded in 24-well plates and incubated at 37°C for 16 to 18 h, and 10-fold serial dilutions of the collected supernatants were added to each well. After absorption for 1 h at 37°C, overlay medium containing 2% FBS and 0.8% methylcellulose was added and incubated at 37°C for 72 h. The overlay medium was discarded and stained with a solution containing 4% formaldehyde and 1% crystal violet in phosphate-buffered saline (PBS) at room temperature for 1 h. The plates were washed with flowing water and dried to count plaques. Viral titers were calculated as the PFU/ml and multiplied by dilution factors.

### Neonatal mouse infection model.

One-day-old specific pathogen-free (SPF) ICR neonatal mice (Beijing Institute of Radiation Medicine) were used to establish the animal model of viral infection. All welfare and experimental procedures were carried out strictly in accordance with the Guide for the Care and Use of Laboratory Animals and the related ethical regulations of the Beijing Institute of Radiation Medicine. All efforts were made to minimize animal suffering. The neonatal mice were randomly divided into five groups, and each group contained three litters (*n* = 8 to ∼10 per litter). One group was inoculated intracerebrally with DMEM (10 μl/mouse) and after 1 h injected intraperitoneally with DMSO (control group). Two groups were inoculated intracerebrally with EV71 CC063 (10^5.5^ CCID_50_ ml^−1^) and after 1 h injected intraperitoneally with FNC or DMSO. Two groups were inoculated intracerebrally with CA16 CC045 (10^2.7^ CCID_50_ ml^−1^) and after 1 h injected intraperitoneally with FNC or DMSO. The survival rates and mean clinical symptoms were monitored daily for 15 days postinfection. The mean clinical symptoms were scored as follows: 0, healthy; 1, lethargy or weakness; 2, wasting; 3, limb tremors; 4, paralysis in hind limb; and 5, moribund or dead. The control mice were healthy throughout the experiments.

### Viral loads in neonatal mouse tissues postchallenge.

After intracerebral inoculation with EV71 CC063 viruses, CA16 CC045 viruses, or culture medium, three experimental neonatal mice and three control neonatal mice were subjected to viral load detection. The EV71 infection and control groups were collected on day 9 postinfection, and the CA16 infection and control groups were collected on day 14 postinfection. All tissues, including brain, lung, spine skeletal muscle, and hind-limb muscle, were weighed individually, homogenized in sterile PBS, disrupted by freeze-thawing, and centrifuged. The samples were treated with TRIzol (Invitrogen) for RNA extraction and viral load determination by real-time PCR, as described previously (42). Viral loads were determined by real-time PCR and expressed as log_10_ copies/mg tissue.

### Histopathological analysis.

A total of 15 mice were sampled, as follows: three normal mice from the mock-infected group (the clinical score of grade 0), three mice from the EV71-infected and DMSO-treated group (the clinical score of grade 4), three mice from the CA16-infected and DMSO-treated group (the clinical score of grade 3), three mice from the EV71-infected and FNC-treated group (the clinical score of grade 3), and three mice from the CA16-infected and FNC-treated group (the clinical score of grade 1). After the mice were anesthetized, lung, brain, spine skeletal muscle, and hind-limb muscle tissues were harvested and immersion fixed with 10% formaldehyde solution for 5 days. Then, all of the samples were dehydrated via an ethanol gradient, clarified through dimethylbenzene, and embedded in paraffin, and 4-μm sections were obtained for hematoxylin and eosin (H&E) staining. Histopathological analysis of the tissues was performed under a light microscope.

### Purification of His-tagged recombinant 3D^pol^.

EV71 3D^pol^ was cloned into the pET28a expression vector with a His tag at the N terminus and expressed in E. coli BL21-derived cells. The 3D^pol^ protein was purified by Ni-column affinity chromatography, and a gel filtration column was connected to an ÄKTA system (General Electric Company, CT, USA). The purity of the resulting 3D^pol^ protein was determined through SDS-PAGE analysis.

### Isothermal titration calorimetry experiment.

ITC experiments were performed by using an ITC200 instrument (MicroCal, Inc., Northampton, MA, USA). The concentration of 3D^pol^ was 100 μM. FNC was diluted with buffer (pH 7.5, 25 mM HEPES, 100 mM NaCl) to yield a solution of 1.0 mM. The sample cell was filled with 3D^pol^ protein solution, and FNC was added by syringe at 25°C. The stirring rate was 750 r/min. All data were analyzed using the Origin 7.0 software.

### Fluorescence-based activity assay for EV71 3D^pol^.

We followed the novel fluorescence-based method for the detection of polymerase activity of EV71 3D^pol^ (52). Reactions were performed in individual wells of black 96-well flat-bottom plates. The standard reaction contained 50 mM Tris-HCl (pH 7.5), 2.5 mM MnCl_2_, 100 μM UTP, 40 μg/ml poly(A), 0.1 mg/ml bovine serum albumin (BSA), and 0.25 μM SYTO 9. Different concentrations of FNC were added to the reaction mixtures. The assay was initiated by the addition of EV71 3D^pol^, and the fluorescence was recorded at 30°C.

### Statistical analysis.

Statistical analyses were conducted using the SPSS 17.0 software. Data are reported as the means ± standard deviations (SDs) of the results from at least two or three independent experiments performed in triplicate. Statistical signiﬁcance was evaluated by Student's *t* test. Significant differences are indicated in figures as follows: *, *P* ≤ 0.05; **, *P* ≤ 0.01; and ***, *P* ≤ 0.001. A *P* value of <0.05 was considered statistically signiﬁcant. “ns” indicates nonsignificant differences.
